# Biomarkers in Gastric Cancer: Blood Biomarkers, Liquid Biopsy, Artificial Intelligence, and Risk-Stratified Care

**DOI:** 10.3390/cancers18172792

**Published:** 2026-08-28

**Authors:** Helen D. Kim, Bryant Morocho, Elliott Lee, Steve Kwon

**Affiliations:** 1 Warren Alpert Medical School, Brown University, Providence, RI 02903, USA; helen_d_kim@brown.edu; 2 Department of Surgery, Division of Surgical Oncology, Roger Williams Medical Center, Providence, RI 02908, USA; bryant.morocho@chartercare.org; 3 Department of Surgery, Roger Williams Surgery and Cancer Outcomes Research and Equity (RWSCORE) Center, Roger Williams Medical Center, Providence, RI 02908, USA; 4 Department of Surgery, Division of Surgical Oncology, Yale University School of Medicine, New Haven, CT 06510, USA

**Keywords:** gastric cancer, biomarkers, liquid biopsy, pepsinogen, artificial intelligence, endoscopic imaging, cancer screening, cost-effectiveness

## Abstract

Background: Gastric cancer is often diagnosed in advanced stages for patients in countries without organized screening processes. Non-invasive biomarkers have been proposed to risk-stratify and identify those who should undergo endoscopy. Methods: We searched PubMed/MEDLINE, Scopus, and Google Scholar for studies published between January 2006 and May 2026 reporting diagnostic accuracy or health–economic outcomes for gastric cancer detection in asymptomatic adults or patients with precursor mucosal lesions. Twenty-five studies met eligibility criteria and were synthesized narratively, with reporting informed by PRISMA-DTA and Ferrari guidance. Results: Systemic inflammatory indices derived from routine blood counts are inexpensive but non-specific, with individual areas under the receiver operating characteristic curve (AUC) between 0.65 and 0.83. Serological markers of mucosal atrophy have a clearer biological basis and carry the review’s only large Western validation dataset but preferentially detect corpus-predominant disease. Liquid biopsy platforms report the highest accuracy, up to an AUC of 0.968, though sensitivity falls to 62.4% for precancerous lesions and to 44% for stage I disease on some platforms. Magnifying narrow-band imaging reported the highest accuracy among the endoscopic modalities reviewed, with an AUC of 0.97. In low-incidence Western settings, one-time serum pepsinogen screening with endoscopy reserved for positive results is cost-effective at USD 4913 per QALY gained. Conclusions: Reported accuracy is encouraging across all four classes of gastric cancer markers but derives mainly from retrospective East Asian case–control cohorts and declines at the earliest stages of disease. The evidence supports using these markers to triage toward endoscopy rather than to diagnose directly. Validation in Western populations remains a gap.

## Simple Summary

Diagnosing gastric cancer early improves five-year survival from 8% to 78%. Endoscopy is accurate but too costly for universal screening in Western countries. This review examines whether simpler diagnostic tests/markers may help identify a high-risk group of people who may benefit from endoscopy. We assessed four groups of markers: inflammation scores from routine blood counts, blood proteins that signal damage to the stomach lining, tests that detect tumor DNA or RNA circulating in the blood, and artificial intelligence applied to endoscopic images. Accuracy across studies was promising, but most data come from retrospective East Asian case–control studies. Performance drops significantly for early-stage cancers and precancerous lesions. We conclude these non-invasive markers are best used to triage high-risk patients for endoscopy rather than replace it, and prospective studies in symptom-free Western populations remain essential.

## 1. Introduction

Delayed diagnosis is the main barrier to improving gastric cancer survival. Early disease is often asymptomatic or produces only non-specific dyspepsia, so in countries without organized screening it is usually found late. In the United States, 32.4% of cases are detected while still localized and 35% are already metastatic at diagnosis [1]. Five-year relative survival is 78.1% for localized disease and 8.1% for distant disease [1]. Stage at diagnosis therefore appears to account for most of the variation in outcome.

Esophagogastroduodenoscopy (EGD) is the clinical gold standard for diagnosis. It allows direct visualization of the gastric mucosa, delineation of mucosal boundaries, and retrieval of tissue biopsies for histopathological confirmation. EGD is also invasive, requires substantial healthcare infrastructure, and costs thousands of dollars per patient, which makes population-wide application financially unsustainable where incidence is low [2,3].

Incidence is unevenly distributed. Annual incidence in East Asia is about five times that of the United States, and 73% of gastric cancers worldwide are diagnosed in Asia compared with 1.5% in the United States [2,4]. Age-standardized incidence in Eastern Asia reaches 32.5 per 100,000 in men, with the highest national rates in Japan (48.1), Mongolia (47.2) and the Republic of Korea (39.7) [5]. Europe is not a single risk stratum and should not be treated as one. Incidence in Central and Eastern Europe is several-fold higher than in Western and Northern Europe, and the highest-incidence European countries, among them Portugal and the Baltic states, approach rates seen in parts of East Asia, while the United Kingdom and the Nordic countries sit at the low end of the European range [4,5]. This heterogeneity matters here, because a screening strategy that is not cost-effective at United Kingdom incidence may be so in Portugal or Estonia, and because the term “Western” conflates populations whose pre-test probability differs by a factor of three or more. China, Korea, and Japan operate endoscopy-based national screening programs. Western health systems have no equivalent. Non-invasive tests that identify who warrants endoscopy are consequently of greater interest in low-incidence settings than in high-incidence ones.

Translational oncology has produced several classes of blood-based markers that signal gastric carcinogenesis before symptoms appear. These include systemic inflammatory indices derived from routine complete blood counts [6], serologic markers of gastric mucosal atrophy and *Helicobacter pylori* infection [7,8], and circulating tumor-derived nucleic acids and epigenetic signatures [9]. Artificial intelligence (AI) and computer-assisted endoscopy have separately improved lesion detection and characterization [10]. The clinical questions these markers are asked to answer are not limited to diagnosis. They span screening of asymptomatic populations, risk stratification to determine who warrants endoscopy, surveillance of established precancerous change, and the health-economic question of whether any resulting strategy is affordable at population scale. This review addresses each in turn. This review summarizes the published evidence across these domains, reports the diagnostic performance and health-economic profile of each class of marker as the source studies reported it, and identifies where the evidence base is strongest and where more evidence is needed.

## 2. Methods

### 2.1. Search Strategy and Database Selection

This review was conducted as a structured narrative synthesis, with reporting informed by PRISMA-DTA (Preferred Reporting Items for a Systematic Review and Meta-Analysis of Diagnostic Test Accuracy) principles and by Ferrari’s guidance for narrative reviews [11,12]. We searched PubMed/MEDLINE and Scopus, and used Google Scholar for gray literature and cross-references. The search covered 1 January 2006 to 15 May 2026. To capture the intersection of clinical findings, biomarkers, and health-system implementation, we used a three-tiered Boolean search string:

PubMed/MEDLINE Search String (*n* = 2016): (“gastric cancer” OR “gastric carcinoma” OR “stomach neoplasm” OR “gastric adenocarcinoma” OR “premalignant gastric lesions”) AND (“biomarker*” OR “systemic inflammation” OR “pepsinogen” OR “cytokine*” OR “interleukin*” OR “systemic immune-inflammation index” OR “SII” OR “neutrophil-to-lymphocyte ratio” OR “NLR” OR “liquid biopsy”) AND (“screening” OR “early detection” OR “early-stage diagnosis” OR “risk stratification” OR “secondary prevention” OR “asymptomatic cohort”);

Scopus Search String (*n* = 2999): same as PubMed/MEDLINE;

Google Scholar Search String (*n* = 119): allintitle: “gastric” (cancer OR adenocarcinoma) (biomarker OR pepsinogen OR inflammation OR NLR OR SII) screening.

PubMed/MEDLINE and Scopus were selected for complementary coverage: MEDLINE provides the most complete indexing of clinical diagnostic literature, while Scopus offers the broader journal base and captures conference proceedings and regional journals that MEDLINE does not index. Scopus overlaps substantially with both EMBASE and Web of Science in oncology and laboratory medicine, which limits the marginal yield of adding them. Google Scholar was searched to capture gray literature and to identify records through forward and backward citation tracking. We acknowledge that omitting EMBASE may have reduced retrieval of European pharmacological and conference literature, and that restricting search terms to English introduces language bias that would most plausibly affect retrieval of Chinese-, Japanese-, and Korean-language reports. Given that the included evidence is already predominantly East Asian, this bias is more likely to have reduced the volume of East Asian literature retrieved than to have distorted the geographical balance of the review in the opposite direction. Both limitations are recorded in Section 8.

### 2.2. Eligibility Criteria and Study Selection

Eligibility followed six criteria categories adapted from the PICOTS framework [13]. Participants were asymptomatic adults aged 18 or older, or patients with precursor mucosal pathology. The index test was a non-invasive inflammatory score, serological panel, or liquid biopsy analyte. The target condition was early gastric cancer or a precancerous lesion. The reference standard was EGD with histopathological verification. Outcomes were diagnostic accuracy or health-economic metrics. Eligible designs were peer-reviewed randomized controlled trials, prospective and retrospective cohort studies, cross-sectional diagnostic accuracy studies, case–control and nested case–control studies, and decision-analytic models.

**Figure 1:**
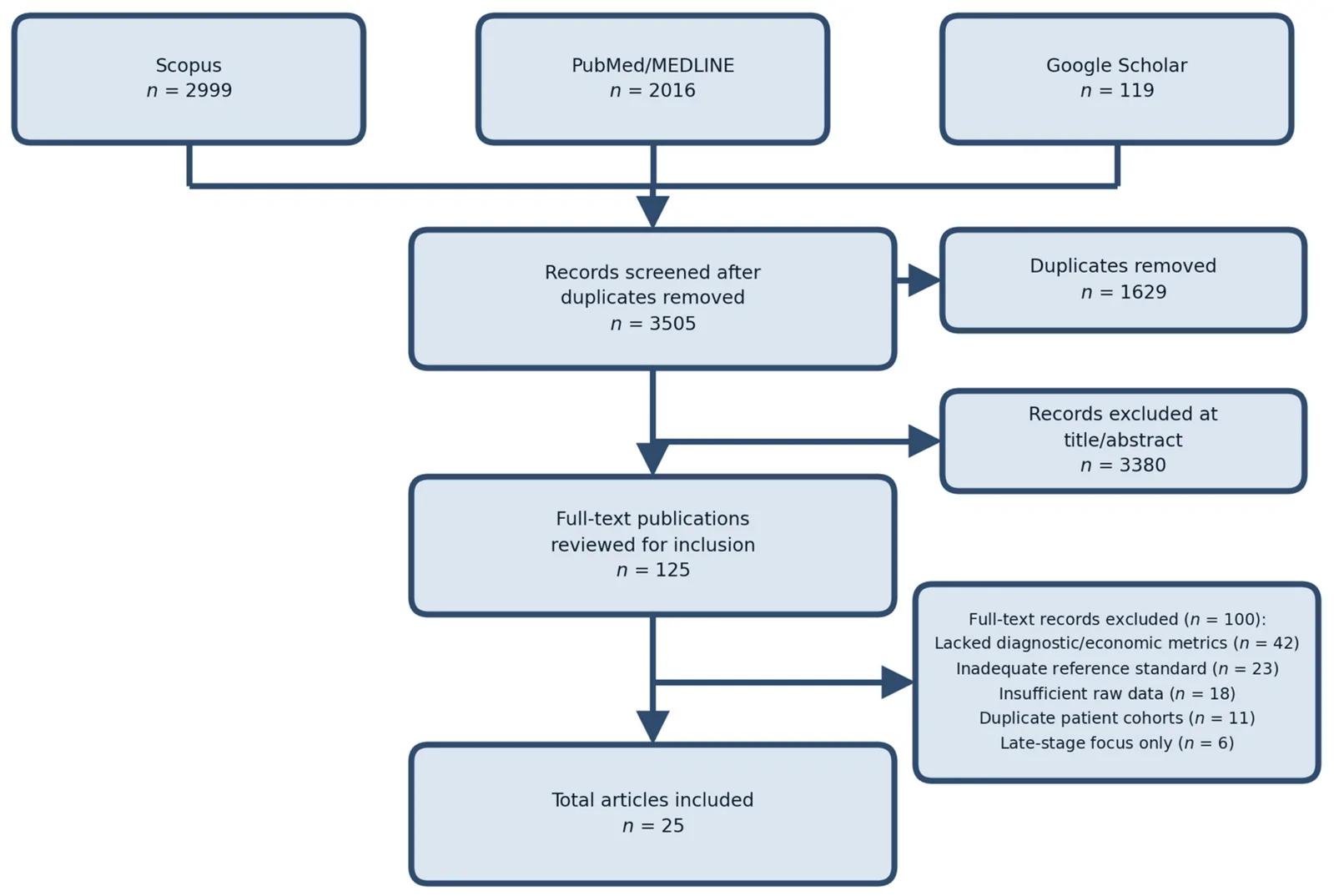
PRISMA-DTA study selection flow diagram. Records were retrieved from Scopus (*n* = 2999), PubMed/MEDLINE (*n* = 2016), and Google Scholar (*n* = 119). After removal of 1629 duplicates, 3505 records underwent title and abstract screening. Full-text review of 125 publications excluded 100 studies for lacking diagnostic or economic accuracy metrics (*n* = 42), inadequate reference standards (*n* = 23), insufficient raw data (*n* = 18), duplicate patient cohorts (*n* = 11), and exclusive focus on late-stage disease (*n* = 6), yielding 25 studies for qualitative synthesis.

Of 5134 records retrieved, 1629 were duplicates. Title and abstract screening excluded 3380 records, leaving 125 articles for full-text evaluation. A further 100 studies were excluded for lacking accuracy metrics (*n* = 42), inadequate reference standards (*n* = 23), insufficient raw data (*n* = 18), duplicate cohorts (*n* = 11), or a focus on late-stage disease (*n* = 6). Twenty-five studies were included for qualitative synthesis (Figure 1): 17 primary diagnostic accuracy studies, three systematic reviews or meta-analyses, four decision-analytic economic models, and one network-biology analysis of circulating microRNA panels. Diagnostic performance is reported throughout as published by the source studies, with discrimination expressed as the area under the receiver operating characteristic curve (AUC). No accuracy estimates were pooled or recalculated across studies. Formal meta-analysis was not undertaken because the included studies differ in index test, analyte, assay platform, threshold, and target condition to a degree that would make a pooled estimate uninterpretable; pooling sensitivity across assays measuring different molecules at different thresholds would produce a number with no clinical referent.

### 2.3. Risk of Bias and Methodological Quality Appraisal

The 17 primary diagnostic accuracy studies were assessed with QUADAS-2 across its four risk-of-bias domains, patient selection, index test, reference standard, and flow and timing, and for applicability of patient selection, judged against this review’s target population of asymptomatic adults in a screening setting rather than against each study’s own stated population. QUADAS-2 evaluates measured diagnostic accuracy and does not apply to synthesized or modeled outcomes, so the three systematic reviews and meta-analyses and the four decision-analytic economic models were not appraised with it; the design of each is identified in the summary table in which its results appears along with its limitations. Assessments were made independently by two authors, with disagreements resolved by discussion. Table 1 reports the results.

Patient selection was at high risk of bias in 8 of the 17 accuracy studies (47%), unclear in 5 (29%), and low in 4 (24%). The high-risk ratings arise almost entirely from two-gate case–control sampling, in which patients with established, often symptomatic, gastric cancer are compared against healthy volunteer controls; in one study the control group was drawn from hospital staff undergoing occupational health examinations [14]. This design maximizes the contrast between compared groups and systematically inflates apparent discrimination relative to what the same test would achieve in consecutive, undifferentiated patients. The index test domain was rated unclear in 11 studies, in most cases because the reported threshold appears to have been selected after the data were seen rather than prespecified, and at high risk in one study in which training and testing sets were drawn at random from a single sample pool with no independent validation [15]. Applicability to an asymptomatic screening population was a great concern in 11 of the 17 studies (65%). Only the nested case–control analysis within the PLCO trial [7] was judged of low concern. Of the five studies rated unclear, two prospective Korean cohorts [16,17] and one prospective multicenter endoscopic study [18] enrolled in a manner that approximates the intended use case.

The reference standard domain was at low risk of bias throughout, since all included accuracy studies used endoscopy with histopathological verification. Flow and timing were rated unclear in most studies because the interval between index test and reference standard was not reported, and at high risk in one opportunistic screening study in which endoscopy was not applied to all participants [19].

**Table 1:** Risk of bias and applicability for the 17 primary diagnostic accuracy studies, assessed with QUADAS-2. L, low; U, unclear; H, high. The reference standard was endoscopy with histopathological verification in all studies. Applicability was judged against this review’s target population of asymptomatic adults in a screening setting.

Study (Reference)	Study Design	Patient Selection	Index Test	Reference Standard	Flow and Timing	Applicability: Patients
Chen K [20]	Retrospective, internal split	H	U	L	U	H
Zhang J [6]	Case–control, 125 vs. 125 healthy	H	U	L	U	H
Fang T [21]	Retrospective	U	U	L	U	H
Zheng J [14]	Retrospective; staff controls	H	U	L	U	H
Wu HM [22]	Retrospective; precancerous comparator	U	U	L	U	U
In H [7]	Nested case–control (PLCO)	L	L	L	U	L
Choi Y [23]	Retrospective	U	U	L	U	H
Lee SY [16]	Prospective, consecutive	L	L	L	L	U
Lim SH [17]	Prospective cohort, PSM	L	L	L	U	U
Lin Z [24]	Case–control, three groups	H	U	L	U	H
Wang Y [25]	Cross-sectional, gastric juice	H	U	L	U	H
Sui S [26]	Retrospective case–control	H	U	L	U	H
Zhu L [27]	Clinical validation	U	U	L	U	H
Ren J [15]	Case–control, 89 vs. 82	H	H	L	U	H
Long VD [28]	Case–control + external validation	H	L	L	U	H
He X [18]	Prospective multicenter	L	L	L	L	U
Xiang W [19]	Hospital-based cross-sectional	U	U	L	H	U

### 2.4. Stratification by Study Design

Included studies are identified by design throughout tables, and performance estimates should be read in that light. Of the 17 primary accuracy studies, three were prospective in design [16,17,18]. A fourth, the PLCO analysis [7], was a nested case–control study using prediagnostic specimens collected within a prospective randomized trial, a design that avoids reverse causation even though ascertainment was retrospective. The remaining 13 were retrospective. Retrospective case–control studies dominate the inflammatory-index and liquid-biopsy literature, while the prospective evidence is concentrated in the serological class. This asymmetry is material: the highest reported accuracies in this review come from the class with the weakest design profile, and the class with the most robust designs reports the most modest figures. Reported accuracy across classes should not be compared without reference to this difference.

## 3. Systemic Inflammatory and Immunonutritional Biomarkers

Chronic inflammation contributes to gastric carcinogenesis from tumor initiation through progression and metastasis [20]. *H. pylori* infection and environmental exposures such as high salt intake produce a sustained inflammatory response in the gastric mucosa [29], which recruits and activates peripheral inflammatory cells and alters routine blood counts in ways that can be measured cheaply and non-invasively [6]. Table 2 summarizes the individual indices and combined panels that have been evaluated on this basis: the Neutrophil-to-Lymphocyte Ratio (NLR), Systemic Immune-Inflammation Index (SII), Platelet-to-Lymphocyte Ratio (PLR), Prognostic Nutritional Index (PNI) and Systemic Inflammation Response Index (SIRI).

Each cell line contributes differently. Reactive oxygen species released by neutrophils cause DNA damage, and neutrophil-derived vascular endothelial growth factor initiates angiogenesis and supports tumor growth. Lymphocytes mediate tumor immunosurveillance, so reduced lymphocyte counts indicate impaired antitumor immunity. Activated platelets, typically elevated in cancer patients, promote tumor growth and neovascularization through VEGF-integrin release [6]. Elevated neutrophil and platelet counts combined with lymphopenia indicate an immunosuppressive, tumor-promoting state, and the composite ratios are designed to capture it.

**Table 2:** Systemic inflammatory and immunonutritional biomarker panels in gastric cancer.

Panel	Formula	Biological Rationale	Clinical Correlates	Performance	Study Design	Ref.
Systemic Immune-Inflammation Index (SII)	P × N/L	Integrates neutrophil, platelet, and lymphocyte counts Reflects host immune-inflammatory balance	Larger tumor size Advanced TNM stage Lymph node metastasis Serosal invasion Response to perioperative FLOT [30]	Cutoff 438.7: AUC 0.831 (GC vs. healthy) [6,30] Cutoff 685.0: AUC 0.652 (mortality) [6]	Retrospective case–control	[6,30]
Neutrophil-to-Lymphocyte Ratio (NLR)	N/L	Systemic inflammatory drive Relative cellular immunosuppression	Stepwise rise: healthy 1.52 → early GC 2.02 → late GC 2.83 [6]	Cutoff 2.1: AUC 0.821 [6] Cutoff 6.48: sensitivity 92.5%, specificity 71.2% [21]	Retrospective case–control	[6,21]
Platelet-to-Lymphocyte Ratio (PLR)	P/L	Platelet-driven VEGF release Supports angiogenesis and tumor progression	Elevated preoperatively Correlates with stage progression and poor cumulative survival	Cutoff 139.5: AUC 0.783 [6] With NLR + SII: AUC 0.843 (95% CI 0.791–0.885), sensitivity 66.4%, specificity 96.8% [6]	Retrospective case–control	[6]
Prognostic Nutritional Index (PNI)	Albumin (g/L) + 5 × lymphocytes (×10^9^/L)	Host immunonutritional status Not an inflammatory ratio as such	Low values track advanced stage Poor differentiation Lauren diffuse-type Increased surgical morbidity	Lymph node metastasis: OR 0.90 [31]	Retrospective; nodal outcome in diagnosed patients	[31]
Systemic Inflammation Response Index (SIRI)	N × M/L	Adds peripheral monocyte dynamics Reflects local immune evasion	Elevated preoperatively Higher histological grade Advanced TNM stage Deeper tumor invasion	Men: AUC 0.720; women: AUC 0.654 [14]	Retrospective case–control	[14]
Combined NLR + PLR + SII (precancerous vs. GC)	NLR, PLR, SII	Multi-parametric inflammatory assessment Comparator is inflamed mucosa, not healthy controls	Abnormally increased in GC relative to precancerous mucosal conditions	NLR: AUC 0.824 [22] PLR: AUC 0.787 [22] SII: AUC 0.842 [22]	Retrospective; precancerous comparator	[22]
Combined CEA + SII + SIRI + PNI (early-stage)	CEA, SII, SIRI, PNI	Adds tumor antigen to inflammatory and nutritional indices Performance is sex-dependent	Evaluated for early-stage (I–II) vs. healthy controls Stage effect observed in men only	Male, early stage: AUC 0.897 (95% CI 0.875–0.918), sensitivity 86.6%, specificity 80.3% [14] Male, advanced stage: AUC 0.745 [14] Female: AUC 0.893 early, 0.898 advanced [14]	Retrospective case–control	[14]
Multimodal Inflammatory-Nutritional Model	Logit(P) from SII + SIRI + PNI + CEA	Adds age, sex, BMI, CA19-9, SII-to-albumin ratio Controls for non-specific metabolic and inflammatory variation	Lauren classification Histological grade TNM stage Not evaluated prospectively for screening	Training AUC 0.93; validation AUC 0.92 [20] Elevated SII/ALB: OR 2.63 [20]	Retrospective; internal training/validation split	[20]

Abbreviations: ALB, albumin; BMI, body mass index; CA19-9, carbohydrate antigen 19-9; CEA, carcinoembryonic antigen; CI, confidence interval; FLOT, fluorouracil, leucovorin, oxaliplatin, and docetaxel; GC, gastric cancer; GIST, gastrointestinal stromal tumor; L, lymphocyte count; M, monocyte count; N, neutrophil count; NLR, neutrophil-to-lymphocyte ratio; OR, odds ratio; P, platelet count; PLR, platelet-to-lymphocyte ratio; PNI, prognostic nutritional index; SII, systemic immune-inflammation index; SIRI, systemic inflammation response index; TNM, tumor, node, metastasis; VEGF, vascular endothelial growth factor.

Among the single indices, the SII performs best. At a cutoff of 438.7, it distinguishes gastric cancer from healthy controls with an AUC of 0.831, and it correlates with larger tumor size, advanced TNM stage, lymph node metastasis, and serosal invasion. At a higher cutoff of 685.0, it predicts mortality with an AUC of 0.652 (Table 2) [6,30]. SII has also been evaluated as a prognostic marker in patients receiving perioperative FLOT chemotherapy for gastric and gastroesophageal junction cancers [30]. The NLR rises stepwise across disease states, from 1.52 in healthy controls to 2.02 in early gastric cancer and 2.83 in late-stage disease, and yields an AUC of 0.821 at a cutoff of 2.1 [6]. At a considerably higher cutoff of 6.48, it reaches 92.5% sensitivity but only 71.2% specificity [21]. The PLR is the weakest of the three individually, with an AUC of 0.783 at a cutoff of 139.5 [6].

Combining the indices improves discrimination. Applied together, the PLR, NLR and SII yield a diagnostic AUC of 0.843 (95% CI 0.791–0.885) with 66.4% sensitivity and 96.8% specificity, higher than any of the three alone [6]. The SIRI, which incorporates peripheral monocyte dynamics, achieves an individual AUC of 0.720 in men and 0.654 in women [14], a sex difference that persists across the other indices in the same cohort and that any prospective evaluation would need to accommodate. The PNI reflects immunonutritional status rather than inflammation as such. In patients with established gastric cancer, it is inversely associated with lymph node metastasis (OR 0.90, 95% CI 0.85–0.96), and lower values correspond to advanced stage, poor differentiation and Lauren diffuse-type histology [31]. This evidence concerns tumor burden in diagnosed patients rather than detection in undiagnosed ones, and PNI has not been evaluated as a standalone detection marker in a screening population.

Two combined panels have been reported with early-stage detection specifically in mind. A panel evaluating NLR, PLR, and SII together to separate precancerous mucosal conditions from established gastric cancer reported AUCs of 0.824, 0.787, and 0.842, respectively, indicating that these indices retain discriminatory value against inflamed precancerous mucosa and not only against healthy cohorts [22]. A four-marker combination of carcinoembryonic antigen (CEA), SII, SIRI, and PNI achieved an AUC of 0.897 (95% CI 0.875–0.918) with 86.6% sensitivity and 80.3% specificity for early-stage disease in men. Performance varied by sex. In men, the AUC fell to 0.745 for advanced-stage disease, while in women early- and advanced-stage performance was almost identical (AUC 0.893 and 0.898) [14]. Any prospective evaluation would need to stratify by sex.

The most complex model identified in this review, the Multimodal Inflammatory-Nutritional Model, combines SII, SIRI, PNI, and CEA with age, sex, BMI, CA19-9, and the SII-to-albumin ratio. It achieved a training-set AUC of 0.93 and a validation-set AUC of 0.92, and an elevated SII/albumin ratio alone carried an OR of 2.63 (Table 2) [20]. The model is strongly associated with Lauren classification, histological grade, and TNM stage. These correlations are with tumor biology in patients who already carry a diagnosis. No study identified in this review evaluated the model prospectively in an asymptomatic screening population, so whether its discriminative performance holds at screening-level disease prevalence is untested.

Specificity is the main limitation of this class. Inflammatory ratios fluctuate with active *H. pylori* gastritis, metabolic syndrome, and unrelated chronic inflammatory conditions [21]. Local up-regulation of indoleamine 2,3-dioxygenase (IDO1) and kynurenine pathway activation suppress T-cell and NK-cell activity within the gastric mucosa [22,32]. These indices are inexpensive and derivable from tests already ordered in routine care. On current evidence, they function as risk indicators that prompt further evaluation, not as standalone diagnostics.

## 4. Serological and Pathological Biomarkers

The Correa pathway describes the histological progression from normal gastric mucosa to intestinal-type gastric cancer, proceeding through chronic superficial gastritis, atrophic gastritis, intestinal metaplasia and dysplasia, with *H. pylori* infection and dietary factors driving the earlier transitions [29]. Parietal and chief cells are progressively lost as atrophy advances [7]. This degeneration can be tracked non-invasively through the pepsinogen series, Pepsinogen I (PG I), Pepsinogen II (PG II), and the PG I/II ratio (PGR), together with Gastrin-17 (G-17). Table 3 summarizes the serological panels built on these markers.

PG I is synthesized by chief and mucous neck cells in the fundic glands of the gastric corpus and fundus. PG II is produced more diffusely, across the fundic glands, antral pyloric glands, and duodenal Brunner’s glands. Under normal conditions, roughly 99% of secreted pepsinogen enters the gastric lumen and only about 1% enters the systemic circulation. It is this small circulating fraction that is measured, and its concentration reflects the mass and secretory capacity of the mucosa that produced it. As chief and parietal cells are replaced by intestinalized mucosa, serum PG I falls below 70 ng/mL [8]. *H. pylori*-induced inflammation simultaneously stimulates PG II secretion across multiple gastric regions, so the PG I/II ratio declines more steeply than PG I alone [7].

The prognostic weight of a pepsinogen-seropositive profile has been quantified in a Western population. In a nested case–control analysis of the United States Prostate, Lung, Colorectal and Ovarian (PLCO) Cancer Screening Trial, prediagnostic PG seropositivity carried an 8.5-fold risk of gastric cancer overall and an 11.1-fold risk for non-cardia disease. The association was not significant for cardia cancer (OR 2.0, 95% CI 0.3–14.2), which is consistent with pepsinogen reflecting fundic-derived pathology (Table 3) [7].

The currency of this dataset requires comment. PLCO enrolled between 1993 and 2001, and the prediagnostic specimens analyzed therefore reflect a United States population with substantially higher *H. pylori* prevalence than the present one. *H. pylori* seroprevalence in the United States has declined across successive birth cohorts, though unevenly: the decline has acted preferentially on non-Hispanic white adults, among whom seroprevalence was 21.2%, while remaining at 52.0% in non-Hispanic Black adults and 64.0% in Mexican American adults [33]. Because pepsinogen thresholds are calibrated against the prevalence of atrophic gastritis in the population that generated them, a cutoff derived from a higher-prevalence era will not carry the same predictive value today. The direction of the effect is predictable: as the prevalence of *H. pylori*-driven corpus atrophy falls, a fixed pepsinogen threshold identifies a smaller absolute number of true positives while continuing to generate false positives from non-atrophic causes of low pepsinogen, so positive predictive value declines even though sensitivity and specificity are unchanged. The odds ratios reported from PLCO should therefore be read as evidence that the biological association is real in a Western population, not as evidence that the specific thresholds remain optimally calibrated.

Contemporary European evidence is sparse but not absent. A prospective single-center Croatian study applying the conventional thresholds of PG I ≤ 70 ng/mL and PGR ≤ 3.0 to 116 high-risk patients reported 78.1% sensitivity and 90.1% specificity for gastric cancer, with a positive predictive value of 71.4% in that enriched population [34]. The sample is small and the population selected, so the study cannot substitute for population-level validation, but it indicates that pepsinogen thresholds developed in East Asian cohorts retain discriminatory value in a European setting. No large prospective validation of pepsinogen screening in an unselected Western population has been published since PLCO. This is the single most consequential evidence gap identified in this review, because the health-economic case for pepsinogen-triaged endoscopy in the United States [35] rests on accuracy parameters that have not been measured prospectively in a contemporary Western screening cohort.

The ABC method translates these markers into a four-category risk stratification by combining anti-*H. pylori* IgG antibody titers with serum pepsinogen concentrations. Group A is negative for both and indicates healthy non-atrophic mucosa. Group B is *H. pylori* positive and pepsinogen negative, indicating superficial gastritis without corpus atrophy. Group C is positive for both, indicating chronic atrophic gastritis with active infection. Group D is *H. pylori* negative and pepsinogen positive, indicating severe extensive atrophy with spontaneous clearance. Relative to Group A, the odds of developing gastric cancer rise from 2.5 to 4.0 in Group B to 6.0–8.5 in Group C and 10.0–14.0 in Group D, the latter particularly for non-cardia cancer and intestinal metaplasia (Table 3) [7,8]. The classic method underperforms in *H. pylori*-endemic regions, where high mucosal inflammation artificially elevates pepsinogen levels and produces false-negative classifications.

The revised ABC method, marketed as GastroPanel, adds G-17 to improve specificity and to allow topographic characterization of atrophy [23]. In corpus-limited atrophy, loss of acid-producing parietal cells causes achlorhydria, which triggers compensatory G-cell hyperplasia and raises serum G-17 above 30 pmol/L. When atrophy or intestinal metaplasia involves the antrum, G-cell mass is depleted and serum G-17 falls below 1.0 pmol/L. Assay platform affects interpretation. A PG I cutoff of 70 ng/mL and PGR of 3.0 on latex-enhanced turbidimetric immunoassay correspond to PG I of 100 ng/mL and PGR of 5.3 on GastroPanel. At the GastroPanel threshold of PGR ≤ 5.3, sensitivity is 51–59% and specificity 61–66%. Combining a low PGR with *H. pylori*-negative status yields adjusted ORs of 3.36 for gastric adenoma and 2.25 for gastric cancer (Table 3) [23]. A prospective evaluation using a PGR cutoff of 6.15 found the threshold significant across all gastric neoplasms, while G-17 (OR 1.030) reached significance only for adenomas and neuroendocrine tumors [16].

Pepsinogen is conventionally understood as a marker of the intestinal-type pathway, but PG II carries information about diffuse-type gastric cancer (DGC), which largely bypasses the Correa cascade. In a large propensity-score-matched Korean cohort, PG II ≥ 21 ng/mL was an independent risk factor, with an OR of 1.66 for gastric cancer overall, 2.35 for DGC, and 4.24 for early-stage DGC. Risk concentrated sharply in women under 40 who were positive for both *H. pylori* and PG II, in whom the OR reached 25.83 (Table 3) [17]. Co-testing PG II with *H. pylori* serology identifies a subgroup that pepsinogen-ratio strategies alone would not flag.

Adding tumor antigens to a mucosal panel improves discrimination between malignant and benign disease, since pepsinogens and G-17 both fluctuate in benign conditions. In a triple-marker serum panel evaluated for early gastric cancer, the individual markers performed modestly: G-17 AUC 0.671, PG I AUC 0.726, PGR AUC 0.769, and CA72-4 AUC 0.602. Combined detection reached an AUC of 0.883 (Table 3) [24]. The same analysis cautions against relying on CA72-4 in isolation, as standalone serum CA72-4 levels frequently do not differ significantly between tumor groups and healthy controls [24]. Any gain from adding tumor antigens must be weighed against the false-positive burden and incremental cost they introduce.

These markers have also been measured directly within the gastric lumen. Quantification of G-17 and Aldehyde Dehydrogenase 1 (ALDH1), a cancer stem cell marker associated with tumor progression, in gastric juice found both significantly elevated in gastric cancer relative to benign gastritis or gastric ulcer. On binomial logistic regression, both were independent predictors (ALDH1 OR 1.095, 95% CI 1.040–1.154; G-17 OR 1.018, 95% CI 1.007–1.029), and a combined model achieved an AUC of 0.792 (Table 3) [25]. Gastric juice sampling requires endoscopy, so this approach does not substitute for serum testing at the screening stage.

## 5. Liquid Biopsy Technologies and Multimodal Assays

Liquid biopsy for gastric cancer has shifted from mutation-centric assays toward multimodal frameworks that integrate circulating cell-free DNA (cfDNA) concentration, somatic mutation profiles, fragmentomics, methylation patterns, extracellular vesicles (EVs), and circulating non-coding RNAs (ncRNAs) [36]. Circulating tumor DNA (ctDNA) is fragmented, tumor-derived cfDNA released through apoptosis and necrosis, and it exhibits irregular nucleosomal cleavage patterns that distinguish it from background cfDNA. Its short half-life permits real-time monitoring of minimal residual disease. In early-stage disease, however, ctDNA constitutes a trace fraction of total cfDNA, which is a central technical obstacle to using it for screening [36]. Table 4 summarizes the platforms evaluated in this review.

The DESTINEX multicenter study evaluated a PCR-based assay analyzing a proprietary combination of cell-free and exosomal microRNAs isolated from serum, on the rationale that small extracellular vesicles encapsulate and protect these ncRNAs from degradation. It reported the strongest performance of any platform reviewed here, with an AUC of 0.968 and 95.0% sensitivity for detection of early-stage (pT1) tumors [26]. Detection at pT1 is the stage at which the other platforms reviewed here lose sensitivity. The assay depends on a machine-learning classifier that will require cross-cohort platform standardization before the reported performance can be assumed to transfer.

The SPOGIT platform targets promoter hypermethylation of tumor-suppressor genes in plasma cfDNA. Hypermethylation is an early event in the Correa cascade, and differentially methylated regions preserve tissue and chromatin context, which enables tissue-of-origin mapping through a Cancer Signal Origin model. Across gastrointestinal cancers, SPOGIT achieved 88.1% overall sensitivity at 91.2% specificity, with 83.1% sensitivity for early-stage (0–II) disease and 62.4% for precancerous lesions [27]. The decline from 88.1% to 62.4% as the target shifts from established cancer to precancerous change is the figure relevant to screening applications. Markov health-state modeling has projected that population-wide implementation of such platforms could raise five-year survival in gastrointestinal cancer by roughly 27% through stage-shifting, though this is a modeled projection and not a trial result [27].

MCTA-Seq (Methylated CpG Tandem Amplification and Sequencing) enables parallel detection of hypermethylated CpG islands, including DOCK10, CABIN1 and KCNQ5, from a single blood draw, and differentiates CpG island methylator phenotype tumors from colorectal and hepatocellular malignancies. Its performance is strongly stage-dependent: sensitivity is 44% at stage I, 59% at stage II, 78% at stage III and 100% at stage IV, against a specificity of 92% [15]. At 44% sensitivity for stage I disease, the assay would miss more than half of the cases a screening program is designed to find, and its reported application is differential diagnosis and tissue localization rather than frontline detection.

A multi-parametric ensemble model addresses low-tumor-fraction regimes by integrating cfDNA methylation, fragmentomics (tumor-derived fragments averaging about 143 bp against about 167 bp in healthy controls), and somatic hotspot mutation profiling. Validated against non-metastatic gastric cancer with a ctDNA fraction below 1%, it achieved an AUC of 0.87 with 75.9% sensitivity and 92.3% specificity [28]. Its main operational constraint is pre-analytical: collection in standard K2-EDTA tubes causes leukocyte lysis, which releases high-molecular-weight genomic DNA and dilutes the short-fragment circulating pool, so specialized collection tubes and sequencing error-suppression are prerequisites.

Circulating ncRNAs and extracellular vesicles have been evaluated more broadly, drawing on miRNA, circRNA, lncRNA, tsRNA, and encapsulated protein cargo isolated from plasma, saliva, or gastric juice. A four-miRNA gastric classification panel derived through network biology analysis reported 89% sensitivity, 90% specificity, and 87% overall accuracy [37]. This class of marker carries substantial methodological variability, including isolation variability, platform-dependent quantification bias between qPCR and RNA-seq, and susceptibility to inflammatory noise, all of which make cross-study comparison difficult.

Across the platforms in Table 4, next-generation sequencing and multi-omics architectures carry high upfront laboratory costs, and none has yet demonstrated cost-effectiveness against conventional endoscopic screening in a prospective population study. A more fundamental limitation should be stated plainly. The purpose of a screening test is to detect disease at a stage at which intervention changes outcome, which for gastric cancer means precancerous change and stage I disease. Measured against that endpoint rather than against established cancer, the platforms reviewed here do not currently perform adequately: SPOGIT detects 62.4% of precancerous lesions and MCTA-Seq 44% of stage I cancers [15,27]. A test that misses more than half of stage I disease does not fulfill the primary function of a screening test, whatever its aggregate AUC. The DESTINEX result is the exception, with 95% sensitivity at pT1 [26], and it is precisely because it is an exception that it requires independent replication in a cohort not used to train the classifier before it can be regarded as established.

## 6. Computer Vision and Advanced Endoscopic Modalities

When serological or liquid biopsy results indicate elevated risk, endoscopic evaluation follows. Conventional white-light imaging (WLI) endoscopy misses a meaningful proportion of gastric cancers. A systematic review and meta-analysis of 22 studies reported a pooled miss rate of 9.4% (95% CI 5.7–13.1%), rising to 10.0% among patients followed after an initially negative examination, with missed lesions concentrated in the gastric body [38]. Superficial early lesions are difficult to distinguish from chronic inflammatory change, and advanced optical modalities have been developed to address this. Table 5 summarizes their reported performance.

i-Scan virtual chromoendoscopy applies contrast, surface, and tone enhancement to improve visualization of surface topography and mucosal relief. A systematic review and meta-analysis of seven studies evaluating i-Scan for gastric cancer detection reported pooled sensitivity of 84%, specificity of 83%, and overall accuracy of 84% [39]. Magnifying narrow-band imaging (ME-NBI) uses narrow-spectrum blue and green light matched to hemoglobin absorption bands to visualize mucosal microvasculature and neoangiogenesis. A meta-analysis distinguishing cancerous from non-cancerous gastric lesions reported an AUC of 0.97 with pooled sensitivity of 88% and specificity of 96%, the highest performance of any endoscopic modality reviewed here [40].

Computer vision has been integrated into endoscopic workflows to reduce inter-observer variability. Convolutional neural networks (CNNs) perform real-time frame classification and lesion segmentation, and have been evaluated prospectively for diagnosing early gastric cancer during magnifying image-enhanced endoscopy in a multicenter study [18]. Vision Transformers (ViTs) use multi-head self-attention to capture global spatial relationships across a mucosal image rather than local features alone, and have been applied to the detection and characterization of flat, superficial early-stage lesions (Table 5) [41]. Reporting for both architectures is less standardized than for the optical modalities. Published accuracy figures are not consistently accompanied by the sensitivity, specificity, and confidence intervals that would permit direct comparison with i-Scan or ME-NBI, and Table 5 reflects this.

Three issues govern whether these architectures translate into practice, and none is resolved by reported accuracy alone. The first is external validation. Model performance is characteristically measured on images drawn from the same endoscopy units, processor generations, and patient populations that generated the training data, and diagnostic-imaging models degrade when applied across institutions whose equipment, image-capture protocols, or case mix differ from the development setting. Almost all published gastric AI development has used East Asian image libraries, in which the prevalence and morphological presentation of early gastric cancer differ from those of Western populations; a model trained where early lesions are common and endoscopists are experienced in detecting them may not transfer to a setting where they are rare. The second is the difference between standalone accuracy and clinical effect. What matters is not whether the model classifies images correctly in isolation but whether endoscopists assisted by it detect more cancers than endoscopists working unaided, a question answerable only by prospective comparative studies of the kind conducted for [18] and not by retrospective image-set benchmarking. The third is implementation. Real-time computer-assisted detection requires integration with the endoscopy processor, a defined protocol for handling disagreement between operator and algorithm, and a governance route for model updating, none of which is addressed in the accuracy literature. Reporting standards compound the problem: as Table 5 shows, published accuracy figures for these architectures are not consistently accompanied by sensitivity, specificity, and confidence intervals, which prevents direct comparison with the optical modalities and would prevent meta-analysis were one attempted.

Molecular and fluorescence-guided endoscopic imaging, in which exogenous probes targeting tumor-associated antigens generate signal contrast independent of mucosal morphology, represents a further category of optical detection. No study meeting the eligibility criteria of this review reported diagnostic accuracy for molecular imaging in gastric cancer screening or early detection in humans. The modality remains investigational for this indication.

## 7. Health-Economic Profile of Biomarker-Based Screening Strategies

Whether a biomarker reaches clinical use depends on whether the strategy built around it is affordable at population scale, and the economic case differs sharply between high- and low-incidence settings. In each case the relevant comparison is not biomarker testing against no screening, but biomarker-triaged endoscopy against either universal endoscopy or no organized program. Table 6 summarizes the strategies for which health-economic evaluations have been published.

In high-incidence East Asian countries, universal endoscopic screening is itself cost-effective, and biomarkers are not required to justify it [45,46]. A Markov model of the South Korean National Cancer Screening Program found that endoscopy-only screening at a three-year interval up to age 69 was the most cost-effective configuration, with an ICER of KRW 13,354,106 per QALY gained, an organized screening rate of 70.8%, and a 24.1% reduction in gastric cancer mortality [42]. This outperformed double-contrast barium swallow, whose diagnostic sensitivity declined from 26.2% in 2010 to 19.5% in 2019 and which therefore generated higher costs per tumor detected [42].

Population-wide endoscopy is not cost-effective in Western countries with low-to-intermediate incidence, and serological triage changes the calculation there. A patient-level state-transition microsimulation of non-cardia gastric adenocarcinoma in the United States found that one-time serum pepsinogen screening at age 40, with endoscopy reserved for positive results, was cost-effective against no screening at an ICER of USD 4913 per QALY gained, well below the conventional USD 100,000 per QALY willingness-to-pay threshold. The modeled strategy produced a 10.9% relative reduction in lifetime gastric cancer incidence, a 10.8% reduction in mortality, and raised the proportion of localized-stage diagnoses from 30.5% to 33.6% (Table 6) [35]. An earlier and separately parameterized analysis found that restricting serological screening to current smokers at age 50 yielded an ICER of USD 76,000 per QALY compared with USD 105,400 per QALY in the general population [43]. The two models differ in structure and comparator and are not directly comparable, though both indicate that efficiency improves as the tested population is narrowed.

The transferability of the United States ICER estimate depends on three parameters to which the model is sensitive and which carry material uncertainty. The first is *H. pylori* prevalence, which determines both the background rate of atrophic gastritis and therefore the yield of pepsinogen testing, and the fraction of the population in whom eradication would alter subsequent risk. United States seroprevalence has fallen across birth cohorts and varies roughly threefold by racial and ethnic group [33], so a single national prevalence input conceals the fact that the strategy’s efficiency differs substantially between subpopulations. The second is specificity under real-world conditions. Pepsinogen thresholds are established in research settings, but active *H. pylori* gastritis raises PG II and can normalize the pepsinogen ratio in patients who do have corpus atrophy, while proton pump inhibitor use, common and frequently unrecorded in primary care, raises pepsinogen concentrations independently of mucosal status. Both effects push observed specificity below modeled specificity, and the ICER is sensitive to that difference because false positives generate the highest downstream cost. The third is the cost of those false positives. At the prevalence prevailing in an unselected Western population, the overwhelming majority of positive screens are false, and the associated costs include not only the endoscopy itself but the biopsies taken during it, the histopathological assessment of those biopsies, the surveillance intervals triggered by incidental findings of intestinal metaplasia, and the procedural complications that accrue at population scale. Published models capture the procedural cost of the index endoscopy more completely than they capture this downstream cascade. The reported figure of USD 4913 per QALY should therefore be read as an estimate conditional on a specific and favorable parameter set, not as a robust point estimate.

In intermediate-risk settings, the analogous question is which patients with established precancerous change warrant surveillance. Biennial EGD surveillance for individuals with histopathologically confirmed intestinal metaplasia, dysplasia or chronic atrophy was cost-effective in Singapore, with an ICER of SGD 25,949 per QALY against a willingness-to-pay threshold of SGD 46,200 [44]. The stratifying variable here is histological instead of serological, but the underlying logic of reserving endoscopy for a defined high-risk subgroup is the same.

Questionnaire data combined with serology has been evaluated directly as a triage instrument. The Sichuan Gastric Cancer Early Detection and Screening (SIGES) project at West China Hospital developed three scoring systems. Scores A and B derive from an electronic questionnaire covering demographics, family history, and upper digestive tract comorbidities. Score C adds sequential serology, comprising hemoglobin, CEA, and carbohydrate antigen 19-9 (CA19-9). Score C achieved an AUC of 0.903 (95% CI 0.889–0.917), significantly higher than Score A (*p* < 0.001), and improved sensitivity and specificity from 67.2% and 87.5% under Score B to 80.8% and 82.7% (Table 6) [19]. The associated implementation protocol restricts endoscopy to urea breath test-positive individuals aged 40–59 and screens all those aged 60 and over. The authors describe the strategy as cost-effective, but no formal economic model has been published, so its economic profile rests on the reduction in procedures implied by sequential triage.

Whichever strategy is adopted, translating a cost-effectiveness estimate into a functioning program raises operational questions that the economic models do not address: provider compliance with testing protocols, handling of abnormal results within primary care record systems, and patient adherence to referral for endoscopy. Implementation frameworks such as RE-AIM are designed to evaluate these dimensions, and remain largely unapplied to gastric cancer screening specifically [3,47]. The absence of implementation evidence is a distinct gap from the absence of accuracy evidence, and closing the first will not close the second.

## 8. Discussion

The evidence reviewed here spans four classes of non-invasive markers of uneven maturity. Systemic inflammatory indices are the cheapest and most immediately available, since they derive from complete blood counts already ordered in routine care. They are also the least specific, with individual AUCs between 0.65 and 0.83 and a response to any inflammatory stimulus rather than to neoplasia specifically. Combining them raises discrimination to 0.843, and adding nutritional and tumor-antigen markers raises it to 0.897, though that figure applies to men only and has not been reproduced in a screening population [6,14]. Table 7 sets the four classes side by side on accuracy, early-stage performance, cost, available prospective and Western data, and readiness for clinical use.

Serological markers of mucosal atrophy rest on a better-defined biological rationale, because pepsinogen concentrations track a specific histological process instead of generalized inflammation, and they carry the only large Western validation dataset in this review through the PLCO trial [7]. Their principal weakness is topographic: pepsinogen detects corpus-predominant atrophy and performs poorly for cardia cancer, and diffuse-type disease requires PG II and *H. pylori* co-testing [17]. Assay standardization compounds the problem, since the same clinical state yields different numerical thresholds depending on platform [23].

Liquid biopsy platforms report the highest overall accuracy of any non-invasive class reviewed, and the DESTINEX exosomal miRNA assay reached an AUC of 0.968 for pT1 disease, a result that awaits cross-cohort replication [26]. On the other platforms, performance degrades as the target shifts earlier, for the reasons set out in Section 5, so the aggregate figures overstate screening utility. These platforms also carry the highest per-test cost and the most demanding pre-analytical requirements.

ME-NBI reported the highest accuracy of any endoscopic modality reviewed here, with an AUC of 0.97, and computer-assisted systems address the observer variability that produces the 9.4% WLI miss rate [38,40]. Endoscopy is also the resource that these blood-based markers are intended to conserve, so its accuracy does not resolve the access problem.

**Figure 2:**
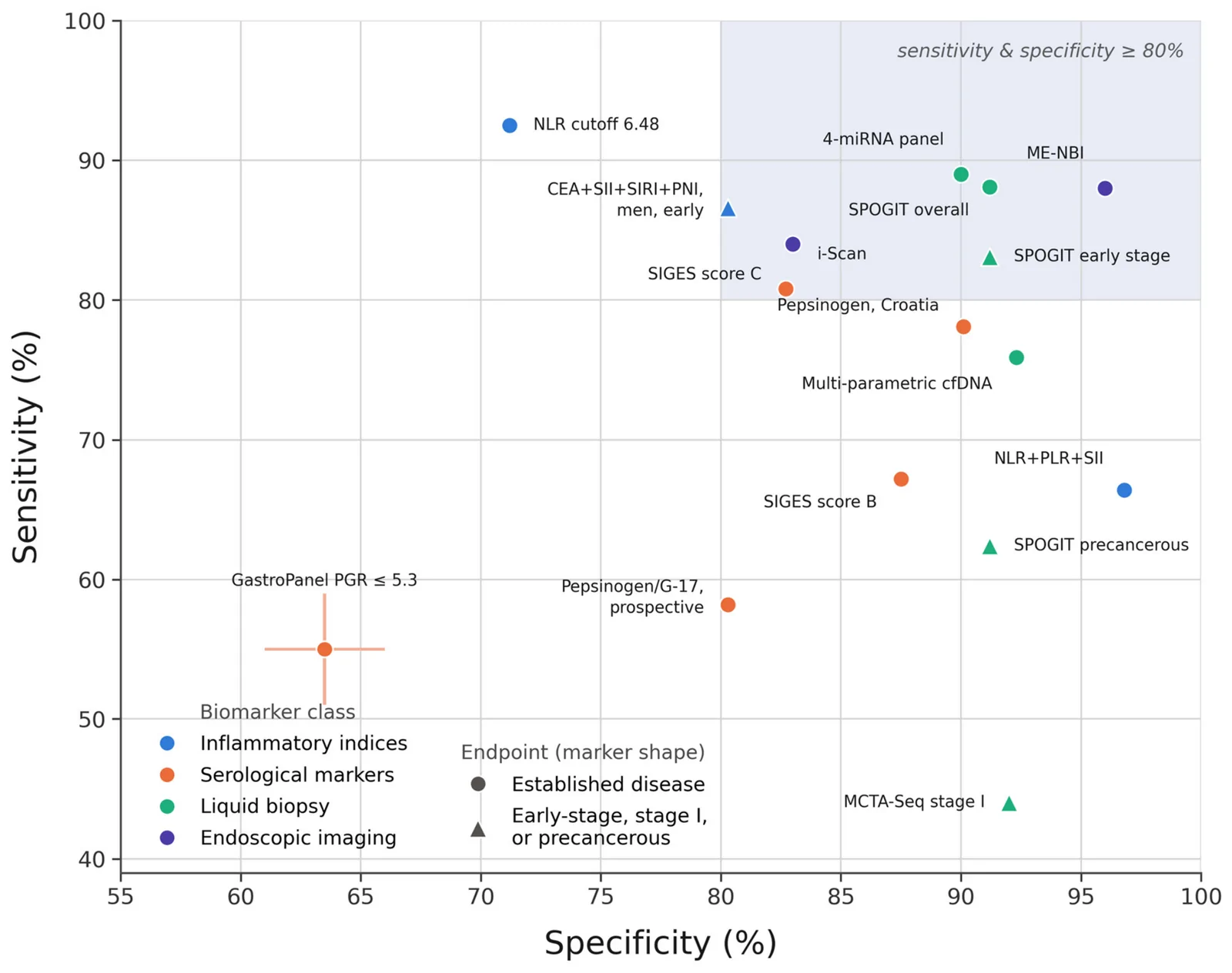
Sensitivity–specificity trade-off across biomarker classes [13,15,17,22,25,31,32,33,34,36,37,46]. Each point is a reported operating point for which the source study gives both sensitivity and specificity, colored by biomarker class. Circles denote performance against established disease; triangles denote an early-stage, stage I, or precancerous endpoint. Cross bars on the GastroPanel point show the reported ranges. The shaded region marks where both sensitivity and specificity reach 80%. Where a platform has been evaluated against progressively earlier disease, sensitivity falls at essentially unchanged specificity (Section 5). The DESTINEX assay is not plotted, since the source reports sensitivity and an AUC but no specificity.

Reported accuracy does not translate directly into screening utility, because predictive value depends on how common disease is in the population tested. SPOGIT reports 88.1% sensitivity and 91.2% specificity [27]. Applied to unselected United States adults over 40, and assuming a prevalence of undetected gastric cancer of approximately 50 per 100,000, those same figures yield a positive predictive value near 0.5%, or about 200 endoscopies for every cancer found [1]. This arithmetic, rather than assay performance, is a principal constraint on blood-based screening in low-incidence Western settings. Figure 2 plots the reported operating points of the markers reviewed here and shows how few of them reach 80% on both axes.

The consequences of that ratio extend beyond the cost of 200 procedures. Upper endoscopy performed in unselected adults over 40 does not return 199 normal examinations and one cancer. It returns a large number of findings of uncertain significance, principally atrophic gastritis and intestinal metaplasia, whose prevalence in Western populations over 50 substantially exceeds that of gastric cancer. Each such finding converts a one-time screening test into an indefinite surveillance commitment, since guidelines recommend interval endoscopy for extensive intestinal metaplasia, and the Singapore analysis indicates that surveillance of this group is itself a cost-bearing program [44]. A screening strategy evaluated only against cancers detected therefore understates its own resource footprint, because it does not count the surveillance population it creates. To these should be added the procedural risks of sedation and perforation distributed across a predominantly disease-free population, and the psychological cost of a positive cancer-screening result in the interval before endoscopy resolves it, a burden borne in this scenario by 199 people for every one in whom it is warranted. None of the economic analyses reviewed here models the surveillance cascade generated by incidental precancerous findings, and this is a specific and addressable gap in the health-economic literature.

Spectrum bias is not the only mechanism inflating these estimates. Verification bias operates wherever the reference standard is applied selectively. In the opportunistic screening protocol evaluated by SIGES, endoscopy was restricted to urea breath test-positive individuals aged 40 to 59 and applied universally only from age 60 [19], so disease status in test-negative younger participants was never established by the reference standard. Where negative index tests are not verified, cancers missed by the index test are not counted as false negatives, and sensitivity is correspondingly overestimated. The effect is not confined to explicitly partial-verification designs: any study recruiting from an endoscopy referral list has already conditioned enrollment on a clinical decision that itself correlates with disease probability. Only designs in which the reference standard is applied irrespective of index test result, or in which complete follow-up establishes outcomes in the unverified, are free of this effect, and among the studies reviewed here only the PLCO analysis [7] and the prospective cohorts [16,17,18] approach that standard.

A related problem limits what cross-platform comparison can mean. The liquid biopsy platforms in Table 4 do not measure the same thing. DESTINEX quantifies exosomal and cell-free microRNA species, SPOGIT and MCTA-Seq interrogate DNA methylation at different genomic targets using different chemistries, and the multiparametric model combines methylation with fragment-length distributions and somatic mutation calling [15,26,27,28]. Their reported AUCs are computed against different control groups, at different disease stage distributions, using classifiers trained on their own data. The numbers are therefore not measurements of a common quantity on a common scale, and any table that places them side by side, including Table 4, should be read with that in mind. The same problem appears in attenuated form among the serological markers, where the identical clinical state yields a pepsinogen ratio of 3.0 on latex-enhanced turbidimetric immunoassay and 5.3 on GastroPanel [23]. Until reference materials and harmonized thresholds exist, the ranking of platforms by reported accuracy should be treated as provisional, and Table 4 is best read as a record of what each platform has claimed under its own conditions rather than as a ranking.

Underlying all of these is a discontinuity between what this literature measures and what would justify a screening program. Every accuracy study reviewed here reports discrimination between people who have gastric cancer and people who do not. No study reviewed here reports whether being screened by any of these markers changes the probability of dying from gastric cancer. Those are different questions, and improvement in the first does not entail improvement in the second: a test can discriminate well and still fail to reduce mortality if the cancers it preferentially detects are those that would not have proved fatal, or if the interval between detectable and clinically apparent disease is too short for detection to alter management. The only mortality estimate in this review comes from an organized endoscopic program, Korea’s 24.1% reduction [42], and it derives from a modality none of these markers replaces. Demonstrating that a biomarker-guided strategy reduces gastric cancer mortality, rather than that it separates cases from controls, is the outstanding research priority for this field, and it is a question that no amount of additional accuracy reporting will answer.

The appraisal reported in Section 2.3 quantifies the concern that this pattern of design creates. Across this evidence base, methodological rigor and reported performance run in opposite directions: the studies enrolling in a manner closest to a screening population are also, with the exception of the endoscopic study, those reporting the most modest figures. That inverse relationship is the most important finding of the appraisal, and it implies that the headline figures quoted in Section 3, Section 4 and Section 5 are more likely to represent ceilings than expectations.

It also explains the shape of the strategies that do appear cost-effective. Each test applied in sequence raises disease prevalence among those who remain eligible, so a subsequent test of fixed accuracy operates at a higher predictive value. The United States pepsinogen model and the SIGES questionnaire-serology protocol both work this way [19,35]. Sequential testing carries a corresponding cost. A patient must test positive at every stage to reach endoscopy, so sensitivity compounds downward and cases missed early are missed permanently. Cost pressure encourages placing the cheapest and least discriminating tests first, where the eligible population is largest, which is also where case loss is greatest. Korea’s organized endoscopic program achieves a 24.1% reduction in gastric cancer mortality [42]. No sequential biomarker strategy assembled from the markers reviewed here has been shown to approach that benchmark, and the published Western economic analyses compare against no screening, not against universal endoscopy.

Several limitations constrain what can be concluded. The majority of performance metrics reported here derive from retrospective, single-center, predominantly East Asian case–control and hospital-based cohorts. These designs carry substantial spectrum bias risk, because they compare patients with established disease against healthy controls instead of evaluating consecutive asymptomatic individuals, and they tend to overestimate the accuracy achievable in a screening population. Clinical cutoffs established in high-*H. pylori*-prevalence East Asian cohorts may not transfer to Western populations that differ in dietary pattern, *H. pylori* prevalence, genetic background, and environmental exposure. Platform-dependent variation in serological assays introduces measurement error across studies, and pre-analytical handling requirements for liquid biopsy are not uniformly reported. Most studies report diagnostic accuracy and not the downstream outcomes, stage shift and mortality reduction, that would justify a screening program. The review was not prospectively registered. Searches were restricted to English-language terms and did not include EMBASE or Web of Science, so relevant non-English and European conference literature may not have been retrieved. Risk-of-bias appraisal was conducted as described in Section 2.3; while it characterizes the quality of the included evidence, it was applied to studies already selected by our eligibility criteria and therefore cannot correct for publication bias in favor of positive diagnostic findings.

The priority for this field is prospective, multi-ethnic validation in asymptomatic cohorts, international harmonization of serological assay thresholds, and health-economic evaluation outside the United States, East Asian, and Singaporean contexts in which the existing analyses were conducted. It bears stating explicitly that not one of the high-performing multimodal models reviewed here, neither the inflammatory-nutritional model reporting a validation AUC of 0.92 [20] nor any of the liquid biopsy platforms, has been evaluated in an asymptomatic, low-prevalence screening population. Every reported figure derives from a setting in which disease prevalence exceeded that of a screening population by two orders of magnitude or more. This is the specific condition under which positive predictive value collapses, and it is therefore the specific condition under which these models remain untested.

## 9. Conclusions

Delayed diagnosis remains the principal barrier to improving gastric cancer survival, and the gap between 78.1% five-year survival for localized disease and 8.1% for metastatic disease defines the value of detecting it earlier [1]. A substantial body of evidence now exists for non-invasive markers across four domains: systemic inflammatory indices, serological markers of mucosal atrophy, liquid biopsy platforms, and computer-assisted endoscopic imaging. Reported diagnostic performance is encouraging in each domain. It derives predominantly from retrospective case–control designs in high-incidence East Asian populations, and it degrades at the earliest and most treatable stages of disease.

In low-incidence Western settings, using a serum marker to select patients for endoscopy is cost-effective at an ICER well below conventional willingness-to-pay thresholds, while universal endoscopy is not [35]. The position the present evidence supports can be stated directly: no single non-invasive biomarker can currently replace endoscopy as the reference standard for gastric cancer diagnosis, and none should be deployed for that purpose. Their defensible role is risk stratification, selecting from an unscreened population the subgroup in whom endoscopy is warranted, and they should be evaluated against that role rather than against diagnostic accuracy in isolation.

Three research priorities follow, and they are specific. First, prospective validation is required in asymptomatic cohorts defined by the characteristics that determine pre-test probability, namely age above 50, *H. pylori* serostatus, first-degree family history, smoking history, and ancestry from a high-incidence region, with recruitment in Western populations of mixed ethnicity and with endoscopic verification applied irrespective of index test result. Second, the comparator in such studies should be biomarker-guided endoscopy against usual care and, where feasible, against universal endoscopy, with stage distribution at diagnosis as the primary endpoint and disease-specific mortality as the definitive one; a randomized design is necessary because the stage-shift benefit claimed for these strategies cannot be distinguished from lead-time bias in an observational one. Third, international reference materials and harmonized thresholds for pepsinogen and for methylation-based assays are prerequisites for pooling evidence across cohorts, and their absence currently prevents meta-analysis of the very data that would justify implementation.

On present evidence, a limited recommendation is defensible for low-incidence countries, offered as expert opinion rather than as a guideline-level conclusion. In Western health systems where population endoscopic screening is not cost-effective, one-time serum pepsinogen testing with *H. pylori* serology, offered around age 50 to individuals carrying additional risk factors and with endoscopy reserved for those screening positive, is the strategy for which the evidence is strongest: it has the only large Western validation dataset [7], contemporary supporting European data [34], prospective evaluation in two cohorts [16,17], and a favorable modeled ICER [35]. The corresponding statement about liquid biopsy is that its current sensitivity for precancerous and stage I disease does not yet support screening use, notwithstanding aggregate AUCs that exceed those of every other class reviewed. Whether the pepsinogen finding holds under prospective evaluation in unselected, multi-ethnic populations will determine whether this recommendation strengthens or is withdrawn.

## Figures and Tables

**Table 3:** Serological and pathological biomarker panels for gastric mucosal atrophy and neoplasia.

Panel	Target Condition	Diagnostic Interpretation	Performance	Study Design	Ref.
ABC Method	Population risk stratification by *H. pylori* status and corpus atrophy	Group A: Hp(−), PG(−)—non-atrophic, uninfected Group B: Hp(+), PG(−)—superficial gastritis Group C: Hp(+), PG(+)—atrophic gastritis with active infection Group D: Hp(−), PG(+)—severe atrophy, spontaneous clearance	Group B vs. A: OR 2.5–4.0 [7,8] Group C vs. A: OR 6.0–8.5 [7,8] Group D vs. A: OR 10.0–14.0 [7,8] Underperforms in Hp-endemic regions (false negatives)	Nested case–control; review of ABC method	[7,8]
PLCO Pepsinogen Panel	Corpus mucosal atrophy (chief and parietal cell loss)	PG I < 70 ng/mL and PGR < 3.0 indicates severe corpus atrophy US population; only large Western validation dataset	Overall GC: OR 8.5 [7] Non-cardia GC: OR 11.1 [7] Cardia GC: OR 2.0 (95% CI 0.3–14.2), not significant [7]	Nested case–control in prospective trial (PLCO)	[7]
Korean PG II and *H. pylori* Panel	Severe mucosal inflammation; diffuse-type pathway	PG II ≥ 21 ng/mL indicates active inflammation Early marker for diffuse-type lesions that bypass the Correa cascade	Overall GC: OR 1.66 [17] DGC: OR 2.35 [17] Early-stage DGC: OR 4.24 [17] Women < 40, Hp(+) and PG II(+): OR 25.83 [17]	Prospective observational cohort; PSM	[17]
L-TIA vs. GastroPanel PGR	Assay platform comparison (turbidimetric vs. ELISA)	PG I 70 ng/mL and PGR 3.0 on L-TIA = PG I 100 ng/mL and PGR 5.3 on GastroPanel Thresholds are not transferable between platforms	PGR ≤ 5.3: sensitivity 51–59%, specificity 61–66% [23] PGR ≤ 5.3 + Hp(−): adjusted OR 3.36 (adenoma), 2.25 (GC) [23]	Retrospective; assay platform comparison	[23]
GastroPanel Topography Panel	Site-specific atrophy (antrum vs. corpus); gastric neoplasms	Low PGR indicates generalized atrophy Elevated G-17 (>30 pmol/L) indicates corpus atrophy with G-cell hyperplasia G-17 < 1.0 pmol/L indicates antral atrophy with G-cell depletion	PGR < 6.15: significant for all gastric neoplasms [16] G-17 (OR 1.030): significant only for adenoma and neuroendocrine tumors [16]	Prospective; consecutive enrollment	[16]
Triple-Marker Serum Panel	Early-stage GC detection and secondary prevention	Combines mucosal secretory function (PG, G-17) with tumor antigen shedding (CA72-4) CA72-4 unreliable in isolation	G-17: AUC 0.671 [24] PG I: AUC 0.726 [24] PGR: AUC 0.769 [24] CA72-4: AUC 0.602 [24] Combined: AUC 0.883 [24]	Retrospective case–control	[24]
Gastric Juice ALDH1 and G-17 Panel	Intraluminal tumor stem cell activity. Requires endoscopy—not a non-invasive screening test	Direct measurement in gastric fluid rather than serum Requires endoscopy; not a screening-stage test	ALDH1: OR 1.095 (95% CI 1.040–1.154) [25] G-17: OR 1.018 (95% CI 1.007–1.029) [25] Combined model: AUC 0.792 [25]	Cross-sectional; endoscopy required	[25]

Abbreviations: ALDH1, aldehyde dehydrogenase 1; CA72-4, cancer antigen 72-4; CI, confidence interval; DGC, diffuse-type gastric cancer; ELISA, enzyme-linked immunosorbent assay; G-17, gastrin-17; GC, gastric cancer; Hp, *Helicobacter pylori*; L-TIA, latex-enhanced turbidimetric immunoassay; OR, odds ratio; PG, pepsinogen; PG I, pepsinogen I; PG II, pepsinogen II; PGR, pepsinogen I/II ratio; PLCO, Prostate, Lung, Colorectal, and Ovarian Cancer Screening Trial.

**Table 4:** Liquid biopsy platforms and multimodal assays for early gastric cancer detection.

Platform	Target and Biosource	Application	Operational Challenges	Performance	Study Design	Ref.
DESTINEX Study	Cell-free and exosomal miRNA signatures Serum	Detection of early-stage (pT1) tumors	Machine-learning classifier dependence Requires cross-cohort standardization	AUC 0.968 [26] Sensitivity 95.0% [26]	Retrospective case–control; multicenter	[26]
SPOGIT Platform (CSO model)	Promoter hypermethylation of tumor-suppressor genes Plasma cfDNA	Early-stage (0–II) GI cancer screening Tissue-of-origin mapping Precancerous lesions	Multi-algorithm machine-learning architecture Demands advanced bioinformatic pipelines	Overall: sensitivity 88.1%, specificity 91.2% [27] Early stage: sensitivity 83.1% [27] Precancerous: sensitivity 62.4% [27]	Clinical validation study	[27]
MCTA-Seq	Genome-scale CpG island hypermethylation (DOCK10, CABIN1, KCNQ5) Peripheral blood	Differential diagnosis (CIMP vs. non-CIMP) Distinguishing GC from CRC and HCC	Low stage I sensitivity Better suited to localization than frontline detection	Stage I: 44% [15] Stage II: 59% [15] Stage III: 78% [15] Stage IV: 100% [15] Specificity 92% [15]	Retrospective case–control; single center	[15]
Multi-Parametric Ensemble Model	cfDNA methylation, fragmentomics (~143 bp tumor vs. ~167 bp healthy), hotspot mutations Plasma	Non-metastatic GC vs. healthy controls Detects ctDNA fraction < 1%	Susceptible to leukocyte lysis Requires specialized collection tubes Requires sequencing error suppression	AUC 0.87 [28] Sensitivity 75.9% [28] Specificity 92.3% [28]	Retrospective case–control + external validation	[28]
Circulating ncRNAs and Extracellular Vesicles	miRNA, circRNA, lncRNA, tsRNA, encapsulated protein cargo Plasma, saliva, or gastric juice	Early-stage detection Minimal residual disease monitoring	High isolation variability Platform-dependent qPCR/RNA-seq bias Susceptible to inflammatory noise	4-miRNA panel: sensitivity 89%, specificity 90%, accuracy 87% [37]	In silico network analysis	[37]

Abbreviations: bp, base pairs; CABIN1, calcineurin binding protein 1; cfDNA, cell-free DNA; CIMP, CpG island methylator phenotype; circRNA, circular RNA; CRC, colorectal cancer; CSO, cancer signal origin; ctDNA, circulating tumor DNA; DOCK10, dedicator of cytokinesis 10; GC, gastric cancer; GI, gastrointestinal; HCC, hepatocellular carcinoma; KCNQ5, potassium voltage-gated channel subfamily Q member 5; lncRNA, long non-coding RNA; MCTA-Seq, methylated CpG tandem amplification and sequencing; miRNA, microRNA; ncRNA, non-coding RNA; pT1, pathologic tumor stage 1; qPCR, quantitative polymerase chain reaction; RNA-seq, RNA sequencing; SPOGIT, Screening for the Presence of Gastrointestinal Tumors; tsRNA, tRNA-derived small RNA.

**Table 5:** Advanced endoscopic imaging and computer-vision modalities for early gastric cancer detection.

Modality	Target and Characterization	Implementation	Performance	Study Design	Ref.
White-light imaging (reference)	Standard endoscopic visualization	Baseline comparator for advanced modalities	Pooled miss rate 9.4% (95% CI 5.7–13.1%) [38] 10.0% after initially negative examination [38] Missed lesions concentrated in gastric body [38]	Systematic review and meta-analysis	[38]
i-Scan Virtual Chromoendoscopy	Surface topography and mucosal relief of suspicious lesions	Contrast, surface, and tone enhancement	Pooled sensitivity 84%, specificity 83%, accuracy 84% [39]	Systematic review and meta-analysis	[39]
Magnifying Narrow-Band Imaging (ME-NBI)	Mucosal microvasculature, neoangiogenesis, lesion border delineation	Narrow-spectrum blue and green light matched to hemoglobin absorption bands	AUC 0.97 [40] Pooled sensitivity 88%, specificity 96% [40]	Meta-analysis	[40]
Convolutional Neural Networks (CNNs)	Real-time frame classification and lesion segmentation	Multicenter prospective evaluation during magnifying image-enhanced endoscopy	Evaluated in real-time clinical use [18]	Prospective multicenter diagnostic study	[18]
Vision Transformers (ViTs)	Detection of flat, superficial early-stage mucosal lesions	Multi-head self-attention capturing global spatial relationships	High reported accuracy; sensitivity, specificity, and CIs not consistently reported [41]	Technical review	[41]

Abbreviations: CI, confidence interval; CNN, convolutional neural network; ME-NBI, magnifying narrow-band imaging; ViT, vision transformer. Reference [41] is cited for architectural description only; it reports no diagnostic accuracy metrics and was not counted among the included studies.

**Table 6:** Health-economic and implementation profiles of gastric cancer screening strategies.

Strategy	Setting	Implementation	Economic Viability	Performance	Study Design	Ref.
Universal endoscopy (NCSP)	High-incidence countries South Korea, Japan	Biennial to triennial EGD for all citizens aged 40+ Upper age limit 69	ICER KRW 13,354,106 per QALY [42] Outperforms barium swallow (sensitivity fell 26.2% → 19.5%) [42]	Organized screening rate 70.8% [42] 24.1% reduction in GC mortality [42]	Markov decision-analytic model	[42]
Targeted serological pre-screening	Low-incidence countries United States	One-time serum PG at age 40 (age 50 for smokers) EGD reserved for positive screens	One-time PG at age 40: ICER USD 4913 per QALY, below USD 100,000 WTP [35] Separate model: smokers at 50, USD 76,000 vs. USD 105,400 general population [43]	10.9% reduction in lifetime GC incidence [35] 10.8% reduction in GC mortality [35] Localized-stage diagnoses 30.5% → 33.6% [35]	Patient-level state-transition microsimulation	[35,43]
EGD surveillance of metaplasia	Intermediate-risk populations Singapore	Biennial or annual surveillance for confirmed IM, dysplasia, or chronic atrophy Stratified by severity of mucosal atrophy	ICER SGD 25,949 per QALY vs. SGD 46,200 WTP [44]	Maximizes localized stage migration [44]	Cost-effectiveness model	[44]
Integrated questionnaire-serology (SIGES)	Opportunistic hospital-based screening China	Score A/B: electronic questionnaire Score C: questionnaire + sequential serology (hemoglobin, CEA, CA19-9) EGD restricted to UBT-positive patients aged 40–59; universal at 60+	Reduces unnecessary procedures through sequential triage [19] No formal cost-effectiveness analysis published [19]	Score C: AUC 0.903 (95% CI 0.889–0.917), *p* < 0.001 vs. Score A [19] Score B: sensitivity 67.2%, specificity 87.5% [19] Score C: sensitivity 80.8%, specificity 82.7% [19]	Cross-sectional + implementation protocol	[19]

Abbreviations: CI, confidence interval; EGD, esophagogastroduodenoscopy; GC, gastric cancer; ICER, incremental cost-effectiveness ratio; IM, intestinal metaplasia; KRW, South Korean won; NCSP, National Cancer Screening Program; PG, pepsinogen; QALY, quality-adjusted life year; SGD, Singapore dollar; SIGES, Sichuan Gastric Cancer Early Detection and Screening Project; UBT, urea breath test; USD, United States dollar; WTP, willingness-to-pay.

**Table 7:** Comparative profile of the four biomarker classes reviewed, summarizing accuracy, early-stage performance, core strength, main limitation, relative cost, available prospective and Western data, evidence maturity and readiness for clinical triage use.

Attribute	Inflammatory Indices	Serological Markers	Liquid Biopsy	Endoscopic Imaging and AI
Representative accuracy	AUC 0.65–0.83 single; 0.84 combined [6,14,22]	AUC 0.67–0.88 [16,24]; OR 8.5 prediagnostic [7]	AUC 0.87–0.97 [26,27,28]	AUC 0.97 ME-NBI [40]
Early-stage performance	AUC 0.79–0.84 vs. precancerous [22]	58.2% sensitivity, prospective [16]	Falls to 62.4% precancerous; 44% stage I [15,27]	Not stage-stratified
Core strength	Derived from blood counts already ordered	Only class with large Western validation [7]	Highest reported discrimination	Definitive; enables biopsy
Main limitation	Non-specific; confounded by gastritis and metabolic syndrome [21]	Topographic—misses cardia and diffuse-type [7,17]	Sensitivity collapses at the stages screening targets	Invasive; the resource being conserved
Relative cost per test	Negligible	Low	High	High
Prospective and Western data	None	PLCO [7]; European cohort [34]; two prospective cohorts [16,17]	None	One prospective study [18]
Evidence maturity	Low	Moderate	Low	Moderate to high
Readiness for triage use	Not ready—adjunct only	Closest to deployment	Investigational	Established reference test

## Data Availability

No new data were created or analyzed in this study. Data sharing is not applicable to this article. The search strings, eligibility criteria, and study selection process are reported in full in Section 2 and Figure 1.
